# Longitudinal associations between adolescent adversity, brain development and behavioural and emotional problems

**DOI:** 10.1016/j.dcn.2025.101646

**Published:** 2025-11-16

**Authors:** Ayla Pollmann, Divyangana Rakesh, Delia Fuhrmann

**Affiliations:** a Department of Psychology, Institute of Psychiatry, Psychology & Neuroscience, King’s College London, London SE5 8AF, United Kingdom; b Department of Neuroimaging, Institute of Psychiatry, Psychology & Neuroscience, King’s College London, London, United Kingdom

**Keywords:** Adverse Adolescent Experiences, White matter microstructure, Fractional Anisotropy, RI-CLPM, Neighbourhood Safety, Family Conflict, Traumatic Experiences

## Abstract

Adolescent adversity could have lasting effects on mental health, potentially through neurodevelopmental changes. This study used a random intercept cross-lagged panel model to examine how adverse experiences, brain development, and behavioural and emotional problems are linked over time in the ABCD study (N ≈ 12.000, USA). We found a positive association between family conflict and behavioural and emotional problems: family conflict was related to increased problems at 10 – 12 years (β = 0.06, p = 0.002), and vice versa. At 12 – 14 years, behavioural and emotional problems were also related to increased family conflict (β = 0.20, p < 0.001). Neighbourhood perception was related to behavioural and emotional problems and white matter microstructure. At 10 – 12 years, low neighbourhood safety was related to lower levels of white matter microstructure (β = −0.04, p = 0.041) and vice versa. It was also associated with more behavioural and emotional problems (β = 0.05, p = 0.015) and vice versa. Behavioural and emotional problems were positively associated with neighbourhood perception for adolescents with more friends (χ²(1) = 9.82, p_Bonf._ = 0.02). These findings underscore the need to consider socio-environmental adversity when examining adolescent brain development and mental health.

## 1 Background

Over two-thirds of children and young people globally encounter adverse experiences (Carlson et al., 2020, Cicchetti, 2016). The term adverse experiences refers to potentially traumatic events, including abuse, neglect, and household dysfunction (Felitti et al., 1998). Previous research suggests that adverse childhood experiences (ACEs) (Felitti et al., 1998) and adverse adolescent experiences (AAEs) (Pollmann et al., 2022, Pollmann et al., 2025) are associated with negative outcomes, including poorer mental health during adolescence and adulthood, later life lower socioeconomic status, and even lower life expectancy (Dube et al., 2003, Felitti et al., 1998, Kessler et al., 2010).

Children undergo biological (e.g., puberty) and social changes (e.g., new social roles) when they transition to adolescence (Andersen and Teicher, 2008, Kolb, 2009, Pollmann et al., 2025). Current definitions of adolescence encompass an inclusive age range of 10–24 years (Sawyer et al., 2018). As adolescents gain autonomy, academic settings, peer and romantic relationships, and neighbourhoods become increasingly central to their lives (Pollmann et al., 2022, Pollmann et al., 2025, Slavich et al., 2019). During adolescence, the wider social environment, including adverse experiences therein, may be especially important for the development of the brain, cognition, and behaviour (Fuhrmann et al., 2015). Therefore, after early childhood, adolescence is thought to be a period of renewed brain plasticity and potentially a sensitive period of development (Fuhrmann et al., 2015). The mechanisms by which adversity shapes brain development and later life outcomes, especially during adolescence, are not fully understood, however.

One potential pathway to linking adversity to poor mental health is neurodevelopment (Short and Baram, 2019). Research suggests a complex interplay between adversity during adolescence, brain development and mental health issues (Ancelin et al., 2021, Tian et al., 2020). In the psychopathology literature on early adversity, it has been hypothesised that childhood trauma is associated with differences in structural and functional brain development, potentially contributing to mental health issues (Tian et al., 2020, Whittle et al., 2024). For example, multiple studies have shown changes in white matter connectivity to mediate links between adverse experiences and mental health symptoms such as depression and substance use (Chahal et al., 2022, Rakesh et al., 2021, Rakesh et al., 2021).

Brain regions are interconnected through white matter tracts. White matter tracts are pathways that transmit neuronal signals. They may influence behaviours dependent on the particular pathways between brain regions (Johansen-berg et al., 2010). White matter microstructure can be studied using Magnetic Resonance Imaging (MRI) techniques (Sporns, 2013), including diffusion tensor imaging (DTI). DTI allows the examination of the microstructural characteristics of white matter tracts using tissue water diffusion (Alexander et al., 2007, Soares et al., 2013). The most frequently utilised DTI metric is fractional anisotropy (FA) (Beaulieu, 2002).

Research has shown associations between lower FA values and various psychopathological conditions, including depression, schizophrenia, and obsessive-compulsive disorder (Bijanki et al., 2015, Jung et al., 2010, Kristensen et al., 2019, Tao et al., 2016). As outlined, this suggests a potential developmental pathway in which white matter microstructure mediates environmental effects on psychopathology (Chahal et al., 2022, Rakesh et al., 2021, Rakesh et al., 2021). While FA is a sensitive measure of microstructural changes (Assaf and Pasternak, 2008), its links to adversity and psychopathology have been inconsistent across studies. Limited research has examined white matter microstructure, particularly as a longitudinal process during adolescence. In this study, we examine, amongst others, whether within-person changes in adversity precede within-person changes in white matter microstructure, which in turn precede within-person changes in psychopathology, consistent with a developmental sequence.

Moreover, adverse experiences have previously been shown to be associated with lower FA levels during childhood (Assari and Boyce, 2021, Dufford and Kim, 2017). Lower integrity of white matter microstructure may reflect lower myelination or axonal injury (Fields, 2008, McCarthy-Jones et al., 2018, Sheikh et al., 2014, Thomason and Thompson, 2011). Childhood maltreatment is associated with differences in microstructural white matter development in children, adolescents and adults, which may increase the vulnerability to mental health issues (De Bellis et al., 2002; H. Huang et al., 2012; Lu et al., 2013; Paul et al., 2008). However, compared to childhood, the effects of adversities on the development of white matter microstructure during adolescence are currently poorly understood. Additionally, the majority of previous research investigating the association between adversity and brain development has been cross-sectional (Assari and Boyce, 2021, De Bellis et al., 2002, Dufford and Kim, 2017, Lu et al., 2013, McCarthy-Jones et al., 2018, Paul et al., 2008, Sheikh et al., 2014), which limits developmental inferences.

Parents remain an important influence in adolescents' lives (Pollmann et al., 2022), including for adolescents’ health and well-being (Guevara et al., 2021). At the same time, increasing conflict within families and care settings, particularly conflict with parental authority, is common as adolescents seek autonomy and develop their identities (Branje, 2018). When conflict involves overt hostility, such as verbal abuse, it becomes distinct from the normal disagreements that occur as adolescents seek autonomy, however (Weymouth et al., 2016). Severe conflict in the family is related to adolescent problems, including mental health issues and social difficulties (Cummings et al., 2015). Cross-sectional research has shown links between familial verbal abuse and individual differences in white matter tract structure (Choi et al., 2009). However, knowledge of the longitudinal pathways between family conflict and white matter development is currently lacking.

Over and above household environments, unsafe neighbourhoods may be particularly important in adolescence compared to childhood. Adolescents typically enjoy greater independence and unsupervised time outside their homes (Sawyer et al., 2018). Previous research has established that the neighbourhood environment is associated with adolescent mental health issues and well-being (McElroy et al., 2019, Midouhas et al., 2021, Mueller et al., 2019). In particular, subjective perceptions of neighbourhood safety (e.g., fear of crime) may be relevant to adolescent behavioural and emotional outcomes (Mueller et al., 2019). Previous research found that perceived unsafe neighbourhoods were related to a decreasing trend in FA between the two imaging timepoints (Pollmann et al., 2023).

Therefore, in this study, we examine two adverse experiences: firstly, family conflict, an adversity within the family context, and secondly, adolescent-perceived neighbourhood perception as a central adverse adolescent experience (AAE) (Pollmann et al., 2022, Pollmann et al., 2025). These two domains capture adversity within both the proximal family environment and the broader community context, reflecting potentially key ecological influences that could be salient during adolescence (Pollmann et al., 2025).

In addition to examining factors that increase the risk for psychopathology, it is important to examine the role of factors that may promote resilience in at-risk adolescents. Recently, researchers have begun to uncover positive, modifiable environmental factors that can support healthy development (Keane and Evans, 2022, Marquez et al., 2023, Traub and Boynton-Jarrett, 2017). For example, adolescents may be especially susceptible to our social environment (Blakemore and Mills, 2014), and peer relations become increasingly important (Brown and Larson, 2009). Friendships, in particular, have been shown to play a role in buffering mental health issues that may arise following adversity in youth (Harmelen et al., 2016, Marquez et al., 2023). This suggests that they could be a key modifiable factor during this developmental stage. During adolescence, the brain undergoes significant development in social cognition, and a network of brain areas coined the “social brain” (Kilford et al., 2016). Within neural circuits related to social cognition, there is significant structural (Mills et al., 2014) and functional maturation (Burnett et al., 2011, Klapwijk et al., 2013), and there could be reciprocal interactions between brain maturation and the social environment, which may be linked to mental health outcomes (Lamblin et al., 2017). However, there is limited research on how and whether peer relationships moderate links between adverse experiences, white matter microstructure, and psychopathology.

To address these gaps in the literature, this study examined how adverse experiences, specifically family conflict or perceived unsafe neighbourhoods, are associated with structural brain development changes and with adolescent behavioural and emotional problems. We also examined if these results varied based on gender and peer relationships. We used random intercept cross-lagged panel models (RI-CLPMs) (Mulder and Hamaker, 2021) to investigate these longitudinal relations with the prospective Adolescent Brain Cognitive Development study (ABCD, N ≈ 12,000). RI-CLPMs are an increasingly popular approach in longitudinal modelling, as they differentiate within-person dynamics from stable, trait-like differences between individuals (as captured by the random intercepts) (Mulder and Hamaker, 2021). Our RI-CLPMs included adversities (i.e., family conflict and low neighbourhood safety), white matter development (i.e., FA) and adolescent behavioural and emotional problems. We also conducted a multigroup analysis to determine group differences dependent on gender differences and peer relations.

We predicted that after controlling for stable differences between individuals, there would be (1) autoregressive/within-person carry-over effects of each variable (values of a variable at timepoint 1 are related to values of the same variable at timepoint 2 and so on). This would be indicative of the continuity of these variables over time. We hypothesise that (2) there will be a cross-lagged effect of adversity on FA (e.g., adversity at timepoint 1 is related to white matter connectivity at timepoint 2 and so on) and from FA to adolescent behavioural and emotional problems. This would indicate, for instance, that adversity impacts neurodevelopmental processes and influences behavioural and emotional outcomes. We predict that (3) the RI-CLPM models will be significantly different between groups of young people with high vs low numbers of friends. This would indicate that social support impacts the relationship between adversity, neurodevelopment, and behavioural and emotional outcomes.

## 2 Methods

### 2.1 Study design

Using the prospective, longitudinal US-based Adolescent Brain Cognitive Development study (ABCD, N ≈ 12.000), we examined neurodevelopmental trajectories after adversity in adolescents and investigated the influence of modifiable environmental factors on mental health. We utilized the ABCD data release 5.1, which included data from baseline and 6-month follow-up (timepoint 1, T1), two-year follow-up (timepoint 2, T2) and four-year follow-up (timepoint 3, T3). The ABCD data are available to be accessed by researchers, please see the ABCD website for further information.

We employed a simplified cross-validation technique, an approach rooted in machine learning (Chollet, 2021, Wongkoblap et al., 2022). This approach can be used to control for researcher degrees of freedom in cases where preregistration of a study is not feasible (Akker et al., 2024), for instance, due to model complexity in Structural Equation Modeling (SEM) approaches. It involves using a smaller subset of the data (in our case, 20 % of the total sample) for initial model development, followed by cross-validation in the remaining larger subset (in our case, 80 %). The 20 % exploratory (or training) sample was randomly selected by an independent researcher (KB) who was otherwise not involved in the data analysis. This subset was used to test model convergence and develop the analysis scripts. The primary analysis was then conducted on the remaining 80 %, the confirmatory sample.

The overall sample included 11,868, and the confirmatory sample included 9495 participants. We used gender in this study to reflect the complex social and cultural dimensions of identity that can influence experiences of adversity and mental health outcomes. The difference between reported gender and biological sex at birth was extremely small (<99.8 % overlap). Individuals who did not identify as male or female (N =10) were coded as NA in the main analysis, as the small sample size prevents further analysis. See Table 1 for sample descriptives of the confirmatory sample at T1 and Table 2 for the overall age and number of participants per measure in the confirmatory sample. For the descriptives of the overall sample, please see Additional File Table 1. The trajectories of adversities and mental health issues per wave can be found in Additional File Fig. 2, Fig. 3. For more information on the cohort, see the ABCD website.

**Table 1:** Descriptives of the confirmatory sample at T1.

**Category**	**Percentage**
Gender	
- Male	≈ 52 %
- Female	≈ 48 %
- Other	< ≈1 %
Ethnicity	
- White	≈ 52 %
- Hispanic	≈ 21 %
- Black	≈ 15 %
- Other	≈ 12 %
Parental Education	
- Bachelor’s degree	28 %
- Master’s degree	19 %
- College	16 %
- Below college degree	17 %

**Table 2:** Average overall age and number of participants per measure and imaging timepoint of the confirmatory sample (N = 9495). Neighb. safety = Adolescents perceived low neighbourhood safety.

**Timepoint**		**Mean age (years)**	**Number of participants per measure**			
			*FA*	*Family conflict*	*Neighb. safety*	*Adolescent problems*
T 1		9.9 (8.9–11.1)	8243	9482	9483	9111
T 2		12 (10.5–14)	5908	8748	8751	8779
T 3		14.1 (12.5–15.7)	2245	3791	3791	3798

There was an overall missingness of 25.9 % across variables. T3 included only a partial release of the ABCD study data, which resulted in higher missingness at T3 compared to T1 and T2. Missingness per measure per timepoint varied between < 0.01 and 78 %. Participants included at T3 were more likely to be male (53 %), white (57 %) and have parents with a bachelor’s degree (30 %); see Additional File Figure 1 and Table 1 for a detailed breakdown of missingness.

### 2.2 Adversity measures

The choice of adversity scales was based on the availability of scales in the ABCD study at the time of data analysis. The use of a RI-CLPM necessitated the selection of variables that were collected at each of the three time points investigated, which was the case for the two adversity measures chosen. Scales were reverse-coded where necessary, such that higher scores reflect higher adversity. See Additional File Figure 2 for the distribution of adversities per timepoint.

#### 2.2.1 Family conflict

Family conflict was assessed using the ABCD Youth Family Environment Scale "Family Conflict Subscale" modified from PhenX (FES). This is a self-reported binary 9-item scale based on Moos and Moos (1994) with moderately high reliability (α= 0.68) (Gonzalez et al., 2021).

#### 2.2.2 Neighbourhood perception – adolescents

Perceived neighbourhood safety was assessed using the ABCD Youth Neighbourhood Safety/Crime Survey Modified from PhenX (NSC). It includes one self-reported item: "My neighbourhood is safe from crime". Response options ranged from 1 ("Strongly disagree") to 5 ("Strongly agree").

#### 2.2.3 Neighbourhood perception – parents

Parent-reported perceived neighbourhood safety was assessed using the Neighbourhood Safety Protocol. This scale includes three items, of which the mean was taken. Items include, for instance, "Violence is not a problem in my neighbourhood", with response options from 1 ("Strongly disagree") to 5 ("Strongly agree"). This Safety from Crime scale has good reliability (α=.77–.82) (Echeverria, 2004, Mujahid et al., 2007).

### 2.3 Adolescent problems and resilience measures

#### 2.3.1 Externalising, internalising, and attention issues in adolescence

Adolescents’ behavioural and emotional problems were examined using the Youth Brief Problem Monitor from the ABCD study, an assessment scale that monitors children's functioning. This scale included items such as "I am unhappy, sad, or depressed". Response options ranged from 0 ("Not true") to 2 ("Very true"). The Brief Problem Monitor for youth is considered to have good reliability (α= 0.88) (T. Achenbach et al., 2011). A sum score was used for this measure, and since the BPM was not administered at baseline, it was included in the model from the 6-month assessment (i.e., the first-time point at which it was administered).

#### 2.3.2 Peer support

Peer support was assessed using the ABCD Other Resilience Scale (YRS). The adolescent-reported survey included two items examining how many close friends the adolescent responder has per gender, which were summed together for this study. Close friends were defined as those you "like spending time with, have fun with, and trust".

#### 2.3.3 Parent-perceived youth mental health

In addition to adolescent perceived mental health, parent perceived youth mental health was assessed as a robustness check using the ABCD Parent-Child Behaviour Checklist (CBCL). This checklist assess behavioral and emotional problems during childhood and adolescence (Achenbach and Ruffle, 2000, Barch et al., 2018).

### 2.4 White matter microstructure

Based on the ABCD study, white matter microstructure across all tracts was examined using the ABCD-specific atlas "AtlasTrack" (Hagler et al., 2009, Holland and Dale, 2011). We assessed brain trajectories by modelling average fractional anisotropy (FA) data within all DTI atlas tract fibres. Investigating all tracts allows for a comprehensive overview of potential global patterns within the brain. The ABCD Data Analytics and Informatics Core (DAIC) equalizes MRI acquisition across sites and establishes standardized image processing and extraction for all measures extraction for all measurements before their release, ensuring the quality control and dependability of the imaging data (Saragosa-Harris et al., 2022). Data was excluded based on the quality control criteria set by the DAIC. See Hagler et al. (2019) for detailed information on the processing steps of the MR data used in ABCD.

#### 2.4.1 DTI data preparation

The exclusion criteria provided by the ABCD release notes were utilized. Participants with *z*-scores above five were excluded (*N* = 8). For full information on inclusion and exclusion criteria in the ABCD, see Hagler et al. (2019). As a robustness check, we conducted motion-adjusted sensitivity analyses. FA values were pre-residualized before the RI-CLPMs using a motion quality control metric, with extreme outliers (>3 SD) excluded prior to residualization.

### 2.5 Data analysis

Data were analysed in R version 4.2.2 with R studio version 2023.01.1. The main analysis scripts have been made openly available (https://brain-adversity-mentalhealth.netlify.app/). Firstly, we conducted a linear mixed model analysis (LMM) using polynomial contrast coding to investigate the development of FA across all brain tracts across the three timepoints.

#### 2.5.1 Random intercept cross-lagged panel model (RI-CLPM)

A random intercept cross-lagged model reflects the relationships between variables across different timepoints. It incorporates "random intercepts" to account for consistent individual differences over time and "cross-lagged effects", which reflect how one variable influences another variable at a subsequent timepoint. The effects within a RI-CLPM are conditional on all other effects in the model. We applied full information maximum likelihood (*fiml*) to model missing data. We used the following metrics to evaluate model fit: the chi-square statistic (χ^2^, good fit indicated at *p* < 0.05), the root-mean-square error of approximation (RMSEA, good fit < 0.06), the standardised root-mean-square residual (SRMR, acceptable fit < 0.08, good fit < 0.05) and the comparative fit index (CFI, good fit > 0.95) (Hooper et al., 2008). See Table 3 for the predicted paths of the RI-CLPM.

**Table 3:** Predictions for the RI-CLPM paths based on Mulder and Hamaker (2021) for adversity (family conflict/neighbourhood safety), white matter microstructure, and adolescent behavioural and emotional problems.

**Random intercept cross-lagged panel model (RI-CLPM)**
The observed score (*OS*) of an individual (*i*) at occasion (*t*) is comprised of the grand mean per occasion (*μ*) plus the between components (random intercepts; *B*) and the within components (difference between expected and observed measurements based on the grand mean and random intercept; *W*). We include three domains: Adversity (A), white matter microstructure (B) and adolescent behavioural and emotional problems (M). Adversity in this study refers to either family conflict or perceived neighbourhood safety. *OS*_*it*_ = *μ*_*t*_*+ B*_*i*_*+ W*_*it*_
(1) **Within-person carry-over effects** - Autoregressive effects: *α*_*t*_ from *W*_*i*__*t−1*_ to *W*_*it*_
• **A (Adversity):** *αA*_*t*_ is positive: An individual who experiences higher adversity (e.g., family conflict) relative to their own expected score is likely to experience a higher amount of adversity relative to their own expected score on the next occasion as well. • **B (White matter microstructure):** *αB*_*t*_ is positive: An individual with lower FA relative to their own expected score is likely to experience a lower FA relative to their own expected score on the next occasion. • **C (Mental health):** *αM*_*t*_ is positive: An individual who has more mental health issues relative to their own expected score is likely to experience more mental health issues relative to their own expected score on the next occasion as well.
(2) **Spillover of the state in one domain into the state of another domain** - Cross-legged effects: *ßAB*_*t*_ from *WT*_*i*__*t−1*_ to *WB*_*it*_ and *ßBM*_*t*_ *from WB*_*i*__*t−1*_ to *WM*_*it*_
• **A (Adversity to white matter microstructure):** *ßAB*_*t*_ is negative: A positive deviation of an individual's expected level of adversity (e.g., family conflict) will be followed by a negative deviation in the individual's expected level of white matter microstructure at the next occasion in the same direction. • **B (White matter microstructure to mental health):** *ßBM*_*t*_ is negative: A negative deviation in the individual's expected level of white matter microstructure will be followed by a positive deviation from an individual's expected level of mental health issues at the next occasion in the same direction.

We used the *lavaan* package (lavaan.org—About lavaan, 2024) in R to analyse three cross-lagged panel models (one per adversity measure) to test the relationship between adversity, brain development and mental health issues. Additionally, we conducted an exploratory multigroup analysis to determine groups based on the number of peers and gender in early adolescence. We determined the groups based on the number of peers at T1 using a median split (high (*N* = 4407) vs low (*N* = 5070) number of friends. A multigroup analysis allows us to test whether subgroups show differences in relationships between adversity, brain development and mental health. Multi-group results were corrected for multiple comparisons by the number of models (12).

In addition, we included gender as an exploratory multigroup analysis to account for known gender differences in brain development trajectories and in the association between adversity and psychopathology. These differences have been documented in previous literature (Bath, 2020, Fuhrmann et al., 2022, Prachason et al., 2023).

## 3 Results

This study investigates the longitudinal relationship between adversity, brain development and adolescent behavioural and emotional problems using RI-CLPMs. The code and output of the analysis can be found here: https://brain-adversity-mentalhealth.netlify.app.

**Fig. 1:**
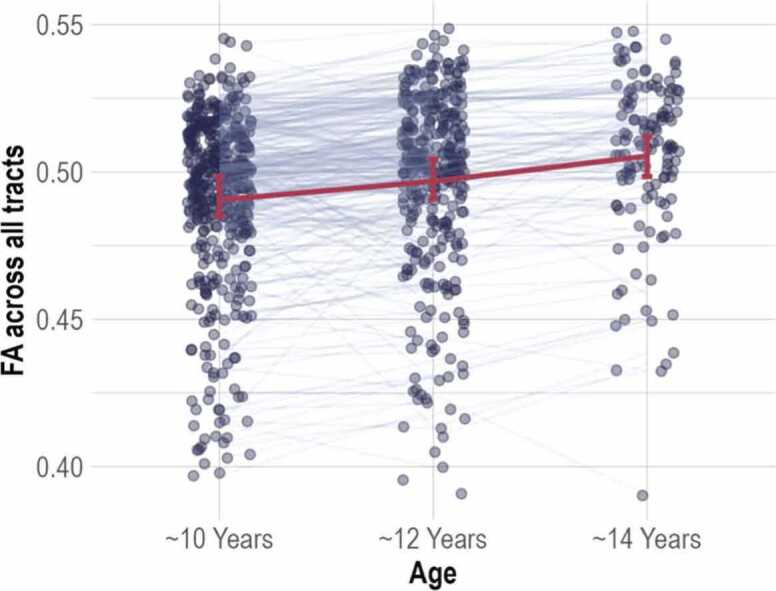
White matter microstructure (FA) development across all tracts. For visual clarity, a random sample of 500 participants from the overall ABCD sample is shown here. The red lines represent the average trend over time and confidence intervals.

We conducted a linear mixed model (LMM) using polynomial contrast coding to investigate the development of FA across all brain tracts for all three timepoints. The results showed that were was a significant linear trend (*β* < 0.01, *t*(9749.42) = 40.16, *p* < 0.001) and a significant quadratic trend (*β* < 0.01, *t*(9513.90) = 3.46, *p* < 0.001. Overall, there was a significant but small increase in FA across all brain tracts, which is in line with previous research (Lebel and Beaulieu, 2011, McLaughlin et al., 2007, Palmer et al., 2021), see Fig. 1**.** Trajectories of friendships and adolescent behavioural and emotional problems across ages can be found in the Additional Files Figure 3.

### 3.1 Family conflict

The RI-CLPM model of family conflict, white matter microstructure and adolescent problems over three timepoints (average ages around 10, 12, and 14 years) showed a good fit (χ²(3) = 28.43, *p* < 0.001, RMSEA = 0.03 [0.02–0.04], SRMR = 0.01, CFI = 0.998).

**Fig. 2:**
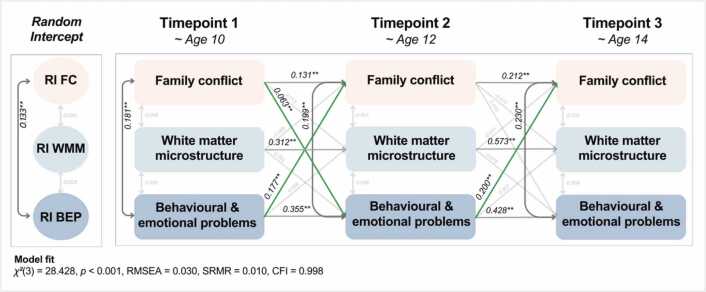
Estimates from the RI – CLPM of family conflict. RI = Random Intercept, FC = Family conflict, WMC = White matter microstructure, BEP = Adolescent behavioural and emotional problems. Light grey lines represent non-significant paths; dark grey lines represent significant paths; green lines are significant positive cross-lagged paths. ** p < 0.01, * p < 0.05.

All autoregressive effects were significant and positive (e.g., for all variables, the previous state positively were associated with the next; for instance, high family conflict scores were associated with high conflict scores at the next timepoint). See Fig. 2 for the path estimates, and details of all statistics can be found here.

The cross-lagged paths indicated reciprocal relationships between family conflict and mental health. Family conflict at T1 was positively associated with adolescent problems at T2 (*β* = 0.06, *p* = 0.002), as were adolescent problems at T1 with family conflict at T2 (*β* = 0.18, *p* < 0.001). Next, adolescent problems at T2 were positively associated with family conflict at T3 (*β* = 0.20, *p* < 0.001). Thus, the results indicate that from age 10–12, family conflict may predict an increase in adolescent problems and vice versa, and from age 12–14, adolescent problems may predict family conflict.

Finally, the random intercepts of these variables were also significantly and positively correlated (*β* = 0.11, *p* < 0.001); see Fig. 2**.** This indicates that in addition to the dynamic effects described above, there were also stable, trait-like associations between family conflict and adolescent problems.

#### 3.1.1 Multigroup – peers

The multigroup results of peer-based resilience for the family conflict model demonstrated a good fit (χ²(6) = 30.42, *p* < 0.001, RMSEA = 0.029 [0.019–0.04], SRMR = 0.01, CFI = 0.999), indicating that the model fits the observed data well across the two groups (high vs. low number of friends). The global differences between the constrained (all cross-lagged paths in the model constrained) and free model (no constrained paths) showed no significant difference in model fit (Δχ²(18) = 17.11, *p* = 0.515), indicating that there are no significant global differences in cross-lagged effects between the two groups. We then tested for local differences by constraining only one cross-lagged path per model at a time but again found no significant differences, indicating that the cross-lagged paths did not significantly differ between groups. The full multigroup results can be found in the Additional Files Table 2.

#### 3.1.2 Multigroup – gender

The multigroup results based on gender for the family conflict model showed a good fit (χ²(6) = 27.74, *p* < 0.001, RMSEA = 0.028 [0.018–0.038], SRMR = 0.01, CFI = 0.999) again, indicating that the model fits the observed data well across the two gender groups. There were no significant global differences across cross-lagged paths (Δχ²(18) = 22.97, *p* = 0.191). There were two significantly different individual paths, but these did not survive correction for multiple comparisons (Table 3 in the Additional Files). Therefore, the cross-lagged paths did not reliably differ between genders.

### 3.2 Neighbourhood perception

**Fig. 3:**
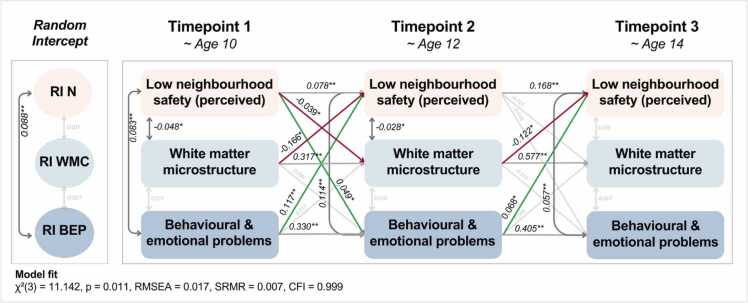
Within-person estimates from the RI – CLPM of perception of unsafe neighbourhoods. RI = Random Intercept, N = Adolescent perceived low neighbourhood safety, WMM = White matter microstructure, BEP = Adolescent behavioural and emotional problems. Light grey lines represent non-significant paths; dark grey lines represent significant paths; green lines are significant positive cross-lagged paths; red lines represent significant negative cross-lagged paths. ** represent p < 0.01, * represent p < 0.05.

The RI-CLPM model of adolescents perceived low neighbourhood safety, white matter microstructure and adolescent problems over three timepoints demonstrated a good fit overall (χ²(3) = 11.14, *p* = 0.011, RMSEA = 0.017 [0.007–0.028], SRMR = 0.007, CFI = 0.999). See Fig. 3 for the path estimates, and details of all statistics can be found here.

All autoregressive effects were significant (e.g., perceptions of low neighbourhood safety were associated with perceptions at the subsequent timepoint). See Fig. 3 for the path estimates, and details of all statistics can be found here.

The cross-lagged paths showed reciprocal relationships between perceived neighbourhood safety and adolescent problems and between neighbourhood safety and white matter structure. Overall, there was a positive relationship between perceived low neighbourhood safety and white matter microstructure (e.g., less safe neighbourhoods were associated with lower connectivity) and a negative relationship with adolescent problems (e.g., less safe neighbourhoods were associated with higher adolescent issues). This is consistent with previous findings indicating that higher FA levels are generally seen in more advantaged groups (Assari and Boyce, 2021, Dufford and Kim, 2017). The coefficients for the perception of low neighbourhood safety at T1 to white matter microstructure at T2 (*β* = −0.04, *p* = 0.041) showed a significant negative association. Low neighbourhood safety at T1 also showed a positive association with adolescent problems at T2 (*β* = 0.05, *p* = 0.015). Conversely, white matter microstructure at T1 was negatively associated with self-reported perceptions of low neighbourhood safety at T2 (*β* = −0.17, *p* = 0.024), and adolescent problems at T1 were positively associated with the perception of low neighbourhood safety at T2 (*β* = 0.12, *p* < 0.001). Next, white matter microstructure at T2 was negatively related to perceived low neighbourhood safety at T3 (*β* = −0.12, *p* = 0.018), while adolescent problems at T2 were positively associated with perceived low neighbourhood safety at T3 (*β* = 0.07, *p* = 0.012). Thus, the results indicate that feeling less safe may be related to lower white matter microstructure levels at age 10–12, while from age 10–14, lower levels of FA may be associated with feeling less safe in one's neighbourhood.

The random intercepts of low neighbourhood safety and adolescent problems were significantly positively correlated (*β* = 0.09, *p* < 0.001). This indicates that in addition to the dynamic effects described, there were stable, trait-like associations between neighbourhood safety and adolescent problems.

#### 3.2.1 Multigroup – peers

The multigroup results of peer-based resilience for the perceived low neighbourhood safety model demonstrated an overall good fit (χ²(6) = 15.22, *p* = 0.019, RMSEA = 0.018 [0.007–0.03], SRMR = 0.008, CFI = 0.999). The global differences between the constrained and free models showed no significant difference in model fit (Δχ²(18) = 24.85, *p* = 0.129). We then tested for local differences; one path remained significant after correcting for multiple comparison. Adolescent problems at T1 were associated with perceived low neighbourhood safety at T2 (Δχ²(1) = 9.82, *p* = 0.002, *p*_*Bonf.*_ = 0.024). This indicates that the cross-lagged path for a high number of friends (*β* = 0.20, *p* < 0.001) and a low number of friends (*β* = 0.04, *p* = 0.262) differ significantly. Thus, adolescent problems at age 10 predict neighbourhood perception at age 12 for adolescents with a higher number of friends but not for those with a lower number of friends. The full multigroup results can be found in the Additional Files Table 4.

#### 3.2.2 Multigroup – gender

The multigroup results based on gender for the low neighbourhood safety model had an overall good fit (χ²(6) = 12.36, *p* = 0.054, RMSEA = 0.015 [0–0.027], SRMR = 0.007, CFI = 1) indicating a good model fit for the observed data across genders. The global differences between the constrained and free models showed no significant differences in model fit (Δχ²(18) = 17.57, *p* = 0.483). The local comparisons were not significant either. The full multigroup results can be found in the Additional Files Table 5.

#### 3.2.3 Parent-perceived unsafe neighbourhood

In addition, we conducted a robustness check analysing parent-perceived levels of low neighbourhood safety rather than young-person-perceived neighbourhood safety (as reported above) to evaluate the potential impact of measurement and reporting issues. The RI-CLPM model of the parent-perceived low neighbourhood safety, white matter microstructure and adolescent problems over three timepoints demonstrated a good fit: all autoregressive effects were significant, but there were no significant cross-lagged paths. See Additional File Figure 4 for further details.

#### 3.2.4 Parent-perceived youth mental health

We included a robustness check to compare youth with parent-perceived mental health issues. For both models (e.g., perception of neighbourhood safety and family conflict), the cross-legged effects between the adverse experiences and white matter microstructure remained largely the same. However, the cross-legged effects between parent-perceived mental health and adversity were mostly non-significant (see Additional File Figure 5). This discrepancy is consistent with prior work showing differences between self- and parent-reports of psychopathology (De Los Reyes et al., 2015, Löchner et al., 2023, Orchard et al., 2019).

#### 3.2.5 Motion adjusted robustness check

Lastly, we conducted motion-adjusted analyses as a robustness check to assess whether findings were robust to residual head motion variability. For both models, the significance of the cross-lagged effects between adverse experiences and behavioural and emotional problems remained unchanged. For the family conflict model, additional negative cross-lagged effects were found between family conflict at T2 and white matter microstructure at T3. Neighbourhood safety showed an additional reciprocal relationship between perception of neighbourhood safety and white matter microstructure at T1-T2 and a reversed effect between white matter microstructure and safety perception at T2-T3. Overall, the motion-adjusted sensitivity analyses suggest robustness of the primary findings (see Additional File Figure 6).

## 4 Discussion

This study investigated the longitudinal interplay between adversity, white matter development, and behavioural and emotional problems in a large adolescent cohort. We aimed to move beyond static, cross-sectional associations by testing a longitudinal pathway and hypothesis: that adversity would predict subsequent changes in both brain development and psychopathology, and that these effects would differ based on social context and gender. This approach allowed us to distinguish between stable, trait-like differences and within-person dynamics over time, providing a more nuanced understanding of how experiences shape developmental trajectories.

The results highlight a complex interplay between adversity, brain development and adolescent behavioural and emotional problems. Each variable demonstrated continuity over time; for example, high family conflict at one time point was consistently associated with high family conflict at subsequent time points. Additionally, different domains were associated with each other over time; for instance, unsafe neighbourhoods during adolescence were related to white matter connectivity. However, family conflict was not associated with white matter connectivity. Additionally, the adversities (family conflict and low neighbourhood safety) were reciprocally linked with adolescent mental health issues. While most longitudinal effects were robust across genders and young people with different numbers of friendships, adolescent problems at age 10 were associated with neighbourhood perception at age 12 only for adolescents with a higher number of friends.

Taken together, these findings show that family conflict and adolescent emotional and behavioural problems may be reciprocally linked, that neighbourhood safety perceptions could be tied to both mental health and brain connectivity, and that peer context could shape these associations. The novelty of this work is in demonstrating how socio-environmental adversity, brain development, and adolescent mental health are linked over time. By highlighting reciprocal relationships between family conflict, neighbourhood safety, and adolescent behavioural and emotional problems, the study underscores the importance of safe neighbourhood environments and broader socio-environmental considerations in understanding adolescent development.

### 4.1 Adversity and brain development

Overall, consistent with prior work (Lebel and Beaulieu, 2011, McLaughlin et al., 2007, Palmer et al., 2021), we found an increase in FA from age 10 to age 14. Increases in FA during this period are likely due to increases in myelination and white matter organization (Lebel and Beaulieu, 2011, McLaughlin et al., 2007, Palmer et al., 2021). Our results indicate a negative association between adversity during adolescence, specifically perceptions of neighbourhood safety, and white matter microstructure: Perceptions of lower neighbourhood safety were related to lower white matter microstructure at a subsequent timepoint between ages 10–12 and vice versa between ages 10 and 14. This aligns with previous research, suggesting that adverse experiences may be linked to lower FA during (late) childhood (Assari and Boyce, 2021, Dufford and Kim, 2017) and that lower perceived neighbourhood safety may be related to a decreasing trend in FA between ages 10 and 12 in specific white matter regions (Pollmann et al., 2023). This study supports the notion that environmental factors, such as a neighbourhood's safety, are linked to young people's brain development and mental health (Burdette and Needham, 2012, De Jesus et al., 2010, Ingoldsby et al., 2006). Our study adds to the literature by longitudinally characterizing such effects in a large, diverse sample of adolescents. These findings highlight the interplay between environmental factors and brain development during adolescence and the individual contributions of different types of adversities.

### 4.2 Behavioural and emotional problems and brain development

Despite previous research finding associations between FA and psychopathology (e.g., Bijanki et al., 2015; Jung et al., 2010; Kristensen et al., 2019; Tao et al., 2016), this effect was not replicated in this study. We do not interpret this as evidence that neurodevelopmental effects of adversity are absent or limited. Rather, it is possible that the relationship between brain development and psychopathology is more complex, involving changes in specific neural pathways that were not captured by our overall FA metric. Additionally, the impact of adversity may depend on its timing, severity, and type (Pollmann et al., 2025), with different forms of adversity operating through distinct neurodevelopmental pathways and potentially manifesting at varying stages of development. Future research should therefore complement global FA measures with more spatially specific approaches, such as tract-specific analysis.

### 4.3 Adversity and behavioural and emotional problems

Our results indicate that adversities (specifically, family conflict and low neighbourhood safety) and adolescent problems were consistently correlated and reciprocally linked; for instance, there was an association between family conflict and adolescent problems throughout early adolescence. This is in line with previous research, indicating that families or care settings remain important during adolescence (Grevenstein et al., 2019, Pollmann et al., 2022).

Our findings are in line with previous research: family conflict and adolescent behavioural and emotional problems are correlated (Fosco and Lydon-Staley, 2020, Herrenkohl et al., 2009; X. Huang et al., 2023). For example, family conflict may predict adolescent mental health problems, such as depression and anxiety (Cummings et al., 2015). Adolescent mental issues could be related to family dynamics (Li et al., 2021) and adolescents with mental health issues report a higher frequency of problematic relationships throughout life (H. Chen et al., 2006). In this study, we included the family conflict items in the ABCD study related to anger, criticism, and physical abuse within the family. While family conflict at age 10 was associated with adolescent problems at age 12, adolescent problems at ages 10 and 12 were associated with family conflict at ages 12 and 14. Thus, not only does conflict within the family predict adolescent behavioural and emotional problems, but adolescent behavioural and emotional problems also predict family conflict. Our study contributes to the current literature by highlighting the reciprocal influence between family conflict and adolescent behavioural and emotional problems, offering longitudinal insights into dynamic family relations.

Neighbourhood safety may become increasingly important during adolescence as adolescents spend their time increasingly outside the family home, for instance, in their neighbourhood environments (Boardman and Saint Onge, 2005). Previous research suggests a reciprocal relation between perceived neighbourhood safety and psychopathology in childhood and adolescence (Meltzer et al., 2007, Midouhas et al., 2021). One potential pathway may be that living in disadvantaged neighbourhoods is associated with heightened levels of stress in the daily lives of adolescents (McBride Murry et al., 2011). In line with this, our results suggest a close link between perceived unsafe neighbourhoods and adolescent mental health issues between ages 10 and 14. Also, adolescent problems at ages 10 and 12 were associated with the perception of low neighbourhood safety at the respective subsequent time points. Thus, adolescent-perceived unsafe neighbourhoods and adolescent problems were correlated and reciprocally linked.

We note, however, that parent-perceived safety did not show the same effects as adolescent-perceived safety. Only the random intercepts of parent-perceived unsafe neighbourhood perception and adolescent problems were correlated, pointing towards stable individual differences. Potential reasons for the differences in parent-adolescent models are that there may be differences in perceptions of neighbourhood safety between age groups (Côté-Lussier et al., 2015) or differences in measurement. Additionally, adversity research has previously shown that the concordance between parent and offspring-reported adversity rates is only moderate (Tajima et al., 2004) and that different measures of neighbourhood safety show different levels of sensitivity (Goldman-Mellor et al., 2016) For instance, Goldman-Mellor et al. (2016) estimated that adolescents who perceived their neighbourhood to be unsafe had a nearly 2.5-fold greater likelihood of mental health issues while living in objectively unsafe areas appears to have no significant impact on the risk of adolescent mental health issues (Goldman-Mellor et al., 2016). Future research should further investigate differences and accuracy in adolescents' and parents' perceptions of neighbourhood safety and the respective influence on adolescent problems.

### 4.4 Friendships and gender

Friendships are a key developmental task during adolescence, and peer support can positively affect youth well-being (Wu and Lee, 2022), as well as mental health issues (Richard et al., 2022, Roach, 2018). Our results showed that the number of friendships during adolescence can be linked to the perception of the safety of the neighbourhood: higher mental health issues were associated with a lower perception of neighbourhood safety for young people with more friends but not for those with fewer friends (adolescent problems at age 10 predict neighbourhood perception at age 12 for adolescents with a higher number of friends). This finding implies that the social environment, particularly the presence of friendships, may play a role in shaping how adolescents view their surroundings. Previous research has highlighted the influence of the social environment on the perception of neighbourhood safety (Salvy et al., 2017). Potentially, adolescents with more friends may spend more time socializing and engaging in activities outside their homes, leading to increased exposure to neighbourhood environments and possibly influencing their perceptions of safety. Additionally, there may be unexamined confounders that could underlie this relationship, such as gene-environment interactions (Esposito et al., 2018, Newbury et al., 2017, Robinette et al., 2018, Strachan et al., 2017). Still, this underscores the importance of considering social factors in understanding how adolescents perceive and interact with their environment. However, further research on the effect potentially detrimental or protective effect of friendships on the perception of neighbourhood safety needs to be conducted.

Despite current research that gender differences may play a role in shaping the effects of adversity, such as abuse, on both brain development and psychopathology (Bath, 2020), our results showed no significant gender differences. Potentially, this may be due to using an overall mental health score. However, internalizing disorders are more common in females, while externalizing disorders are more common in males (Boyd et al., 2015). Future research may, therefore, want to explore specific associations between adversity, brain development and attention, internalizing, and externalizing issues.

### 4.5 Limitations

While we were able to include three timepoints, the last timepoint included only a partial release of data at the time of data access (e.g., T3 includes around ∼27 % of the number of participants of T1). Due to this attrition rate, which resulted in a sample at T3 with higher socioeconomic status, the generalizability of the results may be reduced. To accurately capture brain development trajectories, further research is needed, including more waves of brain development (Parsons and McCormick, 2022). Richer adversity scales and further longitudinal assessments are also essential to increase the understanding of the complex relationships between adversity, the brain and mental health throughout adolescence. It is important to note that our conclusions are based on observational evidence. Potential confounders, such as genetics, may influence neurodevelopmental trajectories (McCrory et al., 2010). Future research could, therefore, build on these findings to test gene-environment interactions. Additionally, the effect sizes of the significant RI-CLPM paths were mostly small (Funder and Ozer, 2019). Still, even small effect sizes may have large and impactful effects on the population level (Carey et al., 2023). A further limitation is our use of a median split to categorise the continuous measure of peer relationships. This approach allowed us to perform a multigroup analysis within the RI-CLPM framework (Mulder and Hamaker, 2021), but it simplifies the dimensional nature of the variable. Future research with a focus on peer relationships is needed and would benefit from using a multi-variate and multi-method approach to modelling peer relationships, to fully capture the potential buffering effects of peer relationships.

Lastly, examining FA across all tracks allowed us to investigate overall patterns of development, which is advantageous for detecting broad, systemic changes in brain structure. Prior developmental neuroimaging studies have demonstrated that examining global network or FA changes reveals important maturational processes, such as increasing brain-wide efficiency, integration, and coherence (Chen et al., 2013; Dennis et al., 2013; Feng et al., 2023; Hagmann et al., 2010; Huang et al., 2015; Lebel and Deoni, 2018; Reynolds et al., 2019). However, we acknowledge that global measures may not capture regionally specific patterns of white matter change. Thus, future work could benefit from complementing our whole-brain approach with tract-specific analyses. In addition, different types of adversity may influence the brain through distinct neurodevelopmental pathways, and effects may manifest at different developmental stages. Future research using multimodal imaging and regionally specific methods will be critical to capture these more nuanced and potentially time-sensitive associations.

### 4.6 Conclusion

This study is one of the first to longitudinally investigate whether individual brain trajectories are linked to adversity and adolescent behavioural and emotional problems. Based on data from the ABCD study, our findings reveal that family conflict, living in unsafe neighbourhoods and lower white matter microstructure may be linked across ages 10 and 14. Adversities were related to and reciprocally linked with adolescent psychopathology. Additionally, friendships may be related to how adolescents perceive their environment, which underscores the importance of considering social factors in adolescent development research. These results enhance our understanding of brain development during adolescence. They also have implications for future research: for instance, the relationship between family conflict and adolescent behavioural and emotional problems suggests that early intervention programs aimed at reducing family conflict may be promising for improving mental health issues in adolescence. Improving adolescent problems may, in turn, improve family cohesion. Additionally, neighbourhoods may be an important environmental factor for adolescent problems and white matter microstructure. This underscores the importance of investigating the effects of community environments. Furthermore, as family conflict and neighbourhood environments may be related to adolescent problems and white matter microstructure, this highlights the necessity of considering broader socio-environmental contexts for understanding adolescent development in research settings. Overall, the relationship between adversity, brain development, and adolescent behavioural and emotional problems is complex. Our findings are shedding light on a period of life during which adverse experiences may shape brain development and mental health.

## CRediT authorship contribution statement

**Ayla Pollmann:** Writing – original draft, Visualization, Validation, Project administration, Methodology, Investigation, Formal analysis, Conceptualization. **Divyangana Rakesh:** Writing – review & editing. **Delia Fuhrmann:** Writing – review & editing, Supervision, Conceptualization.

## Consent for publication

Not applicable.

## Ethics approval and consent to participate

- Most ABCD research sites rely on a central Institutional Review Board (cIRB) at the University of California, San Diego for the ethical review and approval of the research protocol, with a few sites obtaining local IRB approval. All guidelines pertaining to the Declaration of Helsinki were adhered to. Caregivers provided written informed consent and children provided assent for participation in the study. More information on the ABCD study can be found at https://www.sciencedirect.com/science/article/pii/S1878929317302268 and https://abcdstudy.org/about/.

## Funding

- Dr Ayla Pollmann was funded through a PhD research studentship from the Department of Psychology, 10.13039/100009360King’s College London, and the Cusanuswerk. Dr Delia Fuhrmann was supported by an ESRC Secondary Data Analysis Initiative Grant (ES/T015861/1). Dr Divyangana Rakesh received funding from a New Investigator Research Grant from the UKRI Medical Research Council (MR/Z506667/1). For the purposes of open access, the author has applied a Creative Commons Attribution (CC BY) licence to any Accepted Author Manuscript version arising from this submission.
- The ABCD Study® is supported by the National Institutes of Health and additional federal partners under award numbers U01DA041048, U01DA050989, U01DA051016, U01DA041022, U01DA051018, U01DA051037, U01DA050987, U01DA041174, U01DA041106, U01DA041117, U01DA041028, U01DA041134, U01DA050988, U01DA051039, U01DA041156, U01DA041025, U01DA041120, U01DA051038, U01DA041148, U01DA041093, U01DA041089, U24DA041123, U24DA041147. A full list of supporters is available at https://abcdstudy.org/federal-partners.html. A listing of participating sites and a complete listing of the study investigators can be found at https://abcdstudy.org/consortium_members/. ABCD consortium investigators designed and implemented the study and/or provided data but did not necessarily participate in the analysis or writing of this report. This manuscript reflects the views of the authors and may not reflect the opinions or views of the NIH or ABCD consortium investigators. The ABCD data repository grows and changes over time. The ABCD data used in this report came from NIMH Data Archive Digital Object Identifier: 15154/cdn8-s381. DOIs can be found at: https://dx.doi.org/10.15154/cdn8-s381

## Data statement

The data used in this article stem from the ABCD study. The ABCD Study releases curated, anonymized data annually, beginning in 2018, to the research community.

*Data not available / The data that has been used is confidential*

## Declaration of Competing Interest

The authors declare that they have no known competing financial interests or personal relationships that could have appeared to influence the work reported in this paper.

## Data Availability

The authors do not have permission to share data. Data access can be applied for via abcdstudy.org
