# Supplementary material for: Longitudinal associations between adolescent adversity, brain development and behavioural and emotional problems
Source: Dev Cogn Neurosci. 2025 Nov 16;77:101646. doi: 10.1016/j.dcn.2025.101646 (PMC12681635; doi:10.1016/j.dcn.2025.101646)
Supplement: Supplementary file 1 — Supplementary material [file mmc1.docx]

**Additional File**
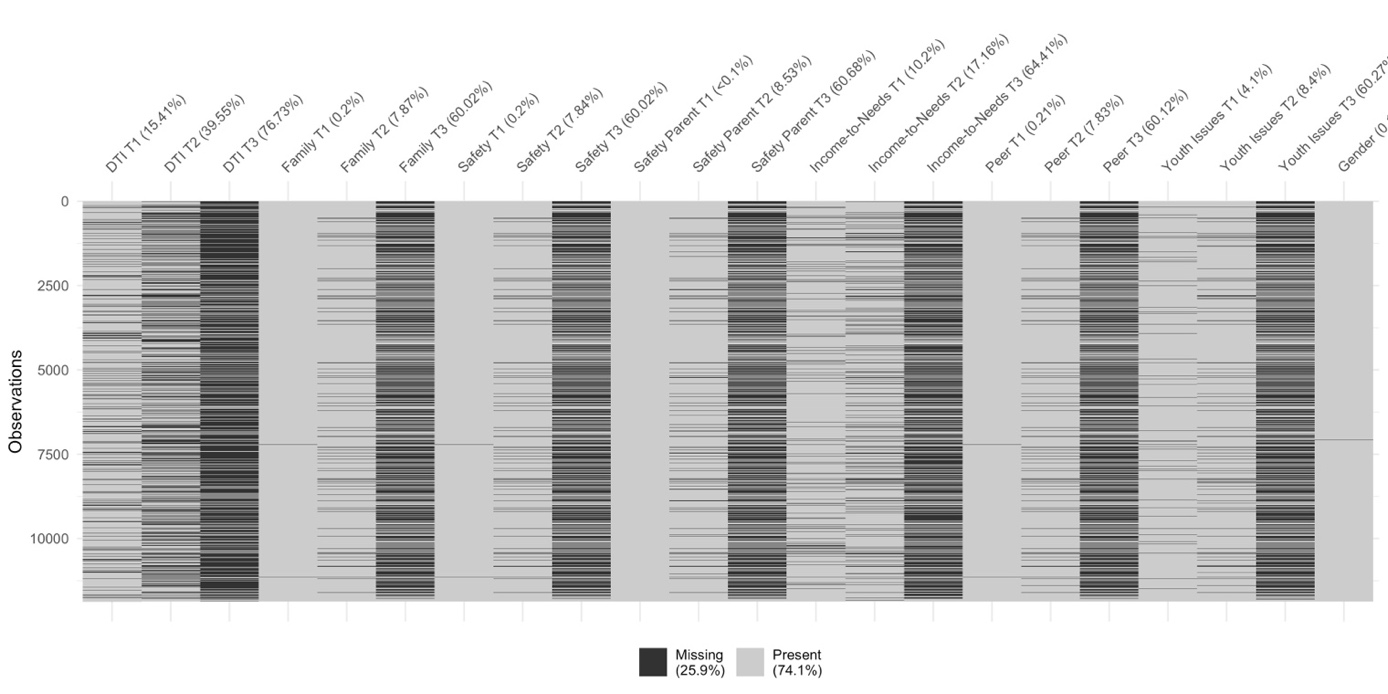
**Figure 1**

Missingness per wave and measure across the full sample


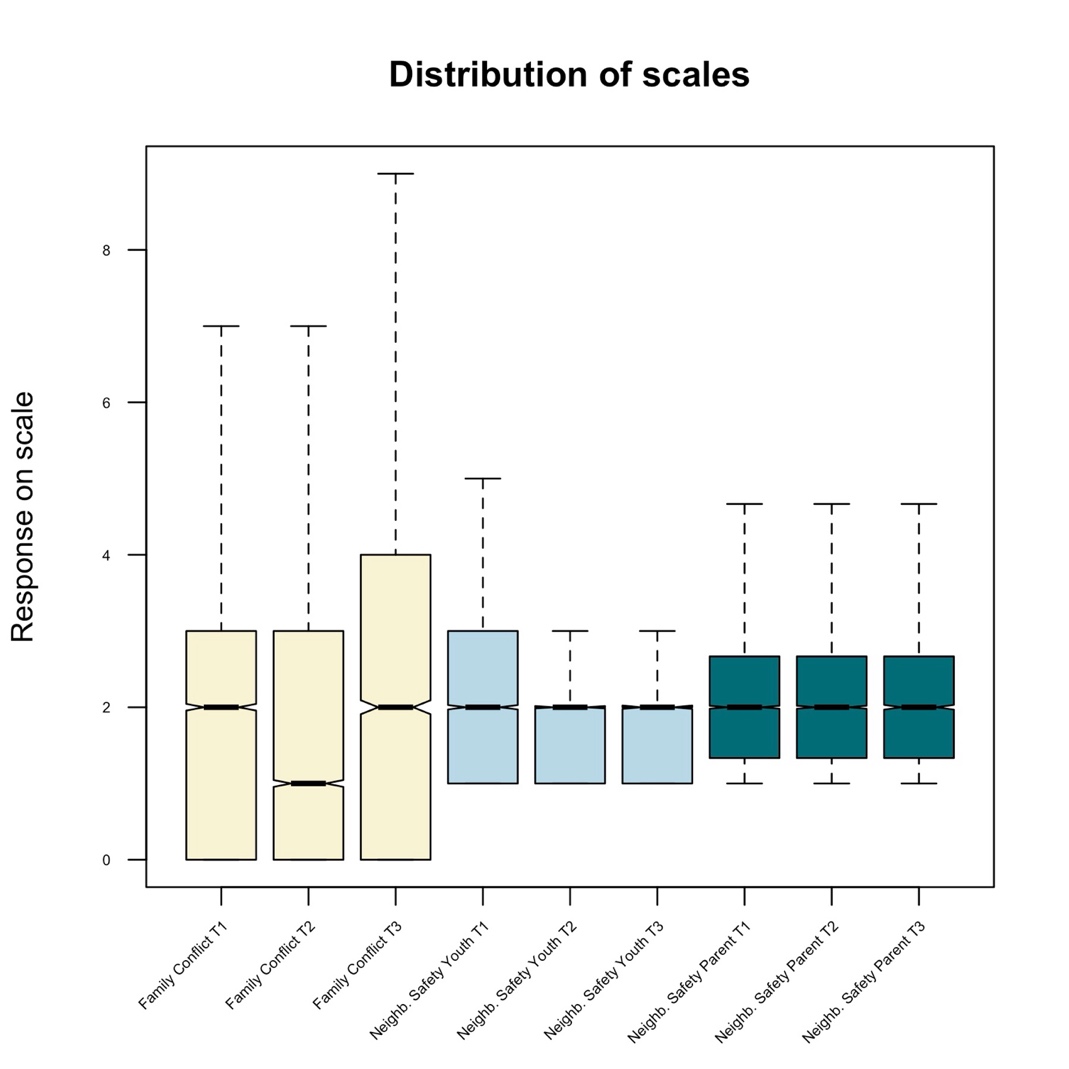


**Figure 2**

Distribution of adversities per time point (T1/T2/T3 = Time point 1/2/3)


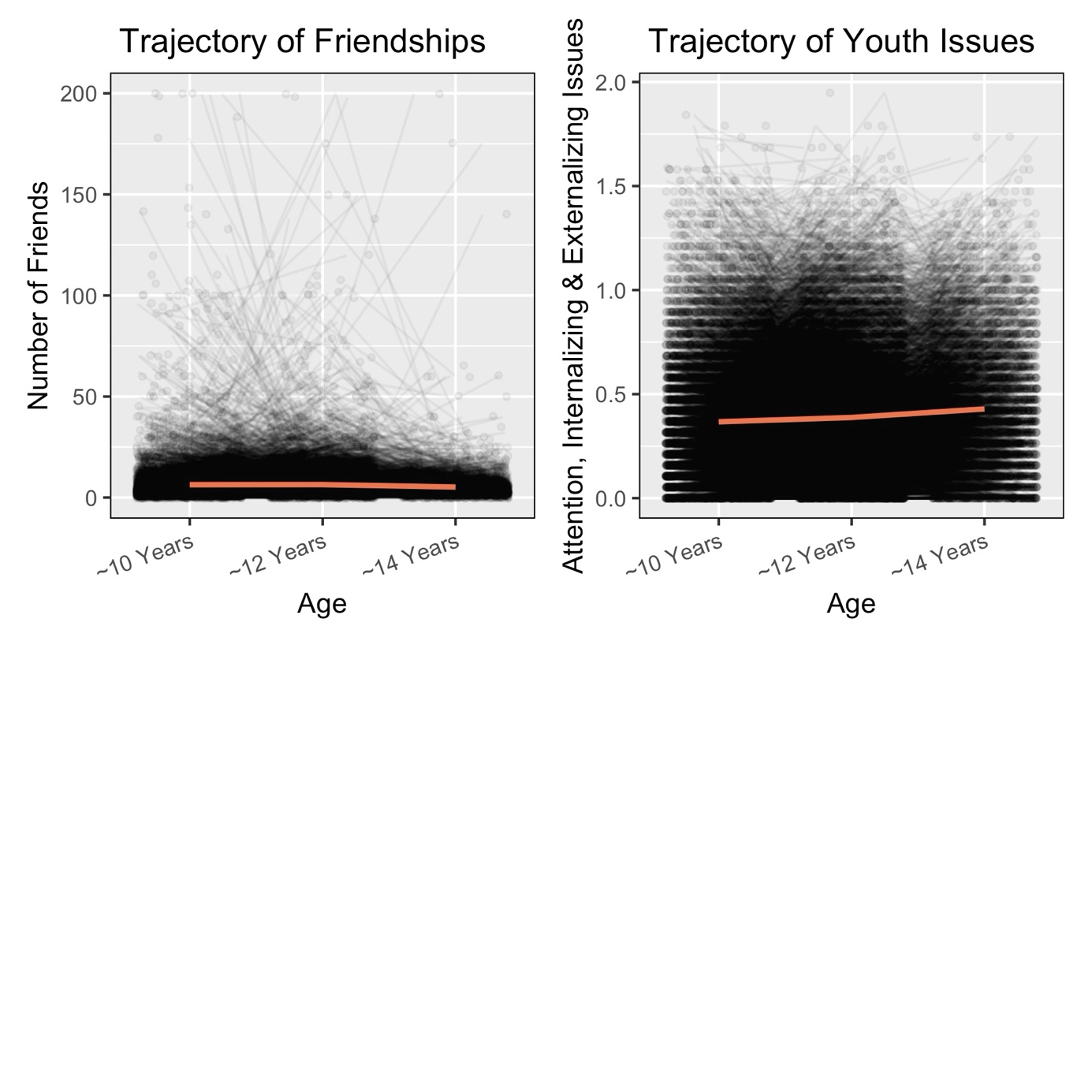


**Figure 3**

Development of friendships and B. & E. Issues within the overall ABCD sample. Red line represents average values per age/wave.

**Table 1**

*Demographics of the overall ABCD sample at baseline & the confirmatory sample at timepoint 3 of those who had FA values at T3*

| **Baseline (overall sample)** | | | | **Timepoint 3 (confirmatory sample)** | |
| --- | --- | --- | --- | --- | --- |
| *Gender* |  | |  |  |  |
|  | Male | ≈52% | | Male | ≈53% |
|  | Female | ≈48% | | Female | ≈47% |
|  | Other | < 0.1% | | Other | < 0.15% |
| *Ethnicity* | | | |  |  |
|  | White | ≈52% | | White | ≈57% |
|  | Hispanic | ≈20% | | Hispanic | ≈20% |
|  | Black | ≈15% | | Black | ≈11% |
|  | Other | ≈13% | | Other | ≈12% |
| *SES (parental level of education)* | | | |  |  |
|  | Masters | ≈19% | | Masters | ≈19% |
|  | Bachelors | ≈28% | | Bachelors | ≈30% |
|  | College | ≈16% | | College | ≈17% |
|  | Bellow College | ≈17% | | Bellow College | ≈14% |

**Parent-perceived unsafe neighbourhood**

We conducted a robustness check analysing parent-perceived levels of low neighbourhood safety, rather than young-person-perceived neighbourhood safety (as reported in the main text) to evaluate the potential impact of measurement and reporting issues. The RI-CLPM model of the parent-perceived low neighbourhood safety, white matter connectivity and adolescent problems over three timepoints demonstrated a good fit (χ²(3) = 19, *p* < 0.001, RMSEA = 0.024 [0.014-0.034], SRMR = 0.009, CFI = 0.999). All autoregressive effects were significant. There were no significant cross-lagged paths. Only the random intercept correlation of perceived low safety and adolescent problems was significant (*β* = 0.07, *p* < 0.001). See ***Figure 4***.


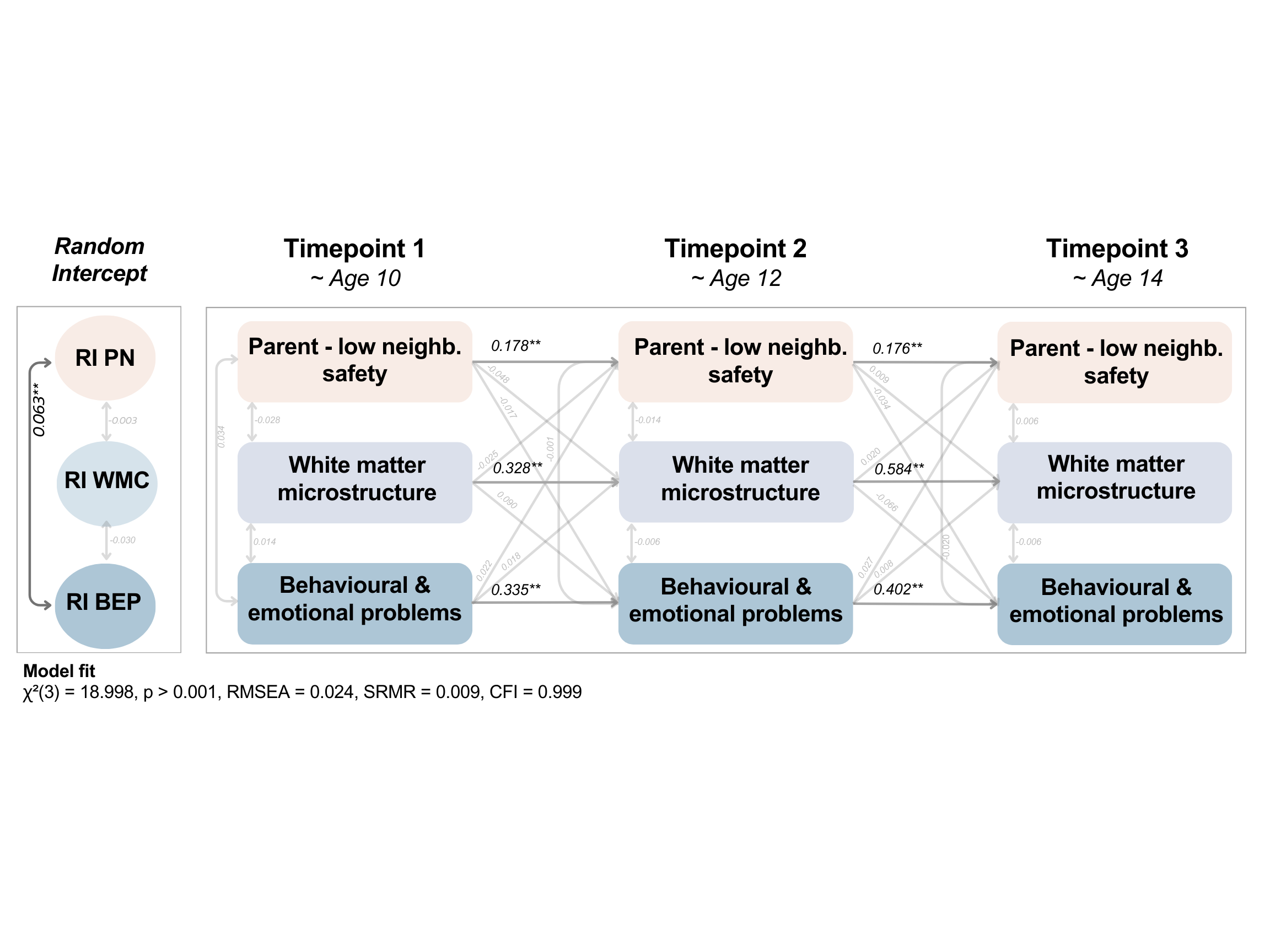


**Figure 4**

Robustness check - within-person estimates from the RI – CLPM of perception of unsafe neighbourhood by parents. RI = Random Intercept, PN = Parent - low neighbourhood safety, WMC = White matter connectivity, BEP = Adolescent behavioural and emotional problems. Light grey lines represent non-significant paths; dark grey represents significant paths. ** represent p < 0.01, * represent p < 0.05.


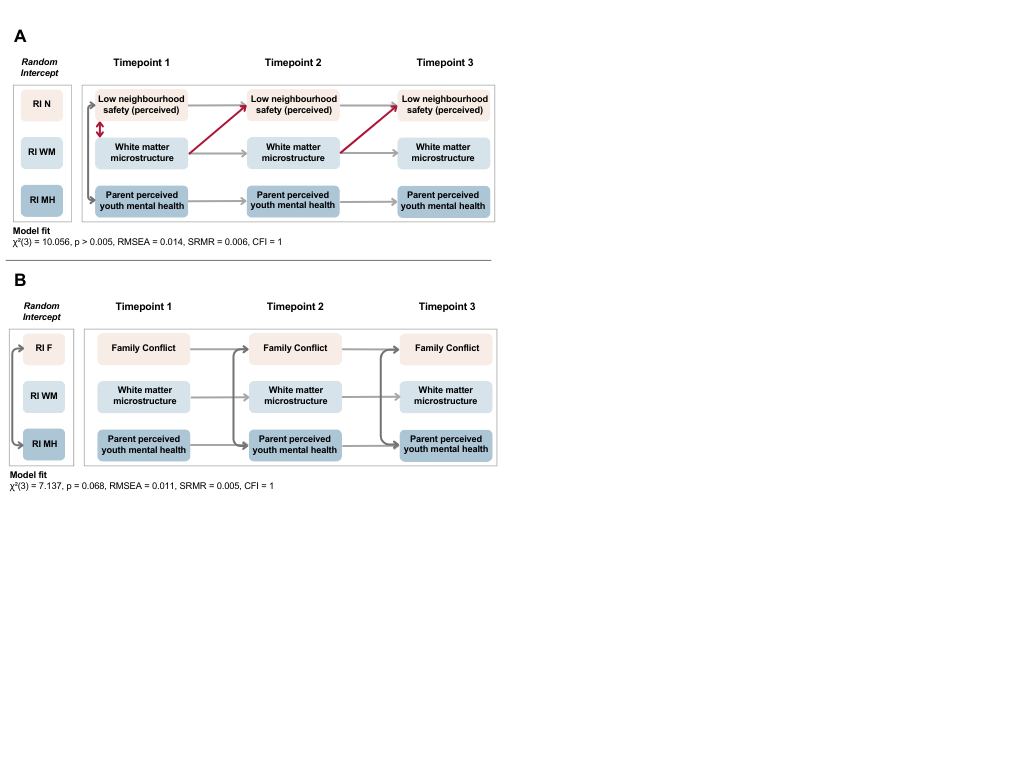


**Figure 5**

Robustness check – analysis with white matter microstructure pre-residualised for head motion. Within-person estimates are shown for the RI–CLPM of perceptions of unsafe neighbourhoods (Figure A) and family conflict (Figure B), and youth mental health. RI = Random Intercept, N = Neighbourhood safety, F = Family conflict, WMM = White matter microstructure, MH = Parent perceived youth mental health. Grey and red lines represent positive and negative significant paths, respectively, at p < 0.05.


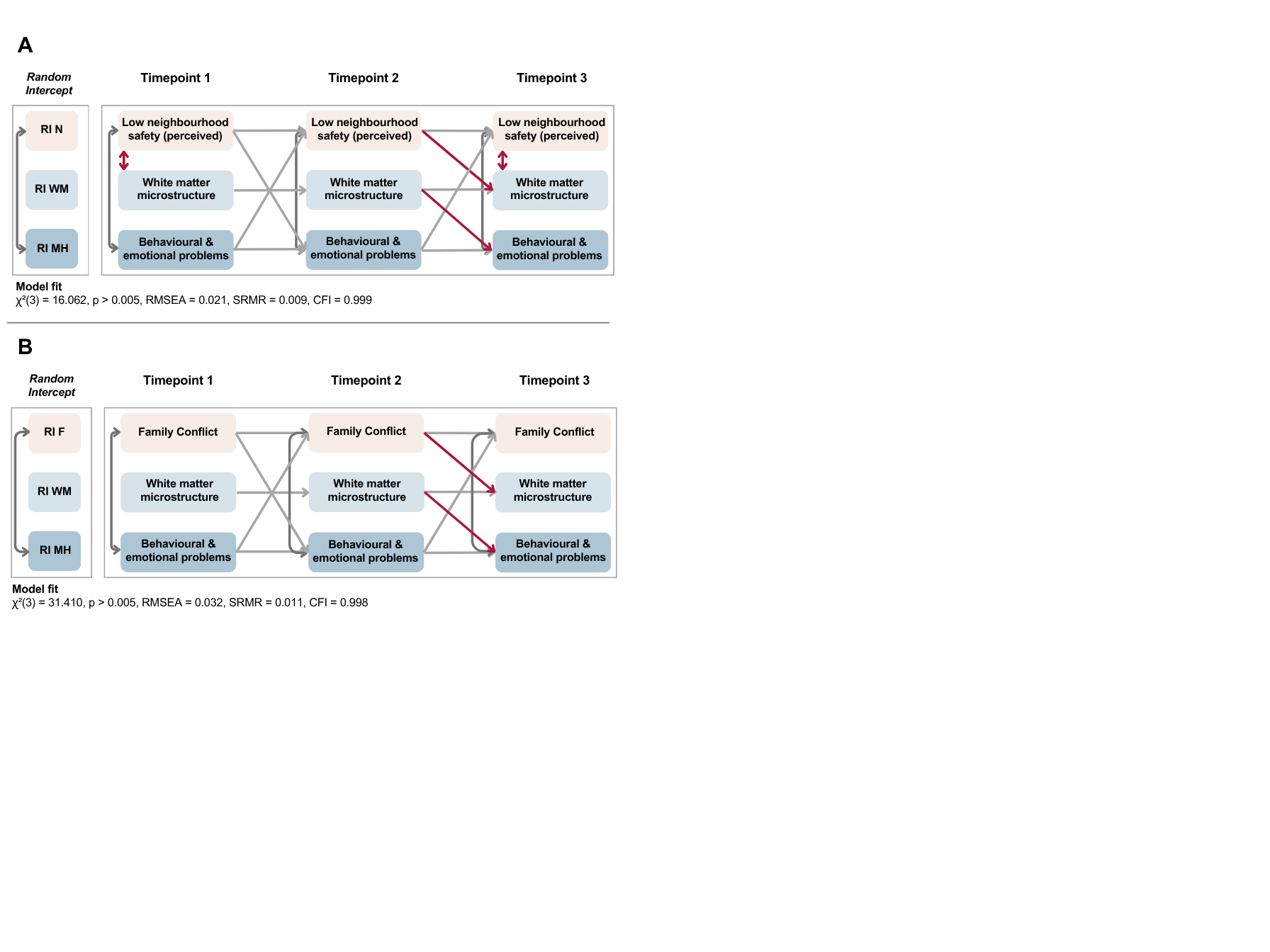


**Figure 6**

Within-person estimates from the RI–CLPM of perceptions of unsafe neighbourhoods (Figure A) and family conflict (Figure B), and youth mental health. RI = Random Intercept, N = Neighbourhood safety, F = Family conflict, WMM = White matter microstructure, MH = Youth mental health. Grey and red lines represent positive and negative significant paths, respectively, at p < 0.05.

**Table 2**

*Multigroup results of constrained paths for family conflict and peers. B. & E. Issues = Behavioural and emotional issues.*

| Constrained Cross-Lagged Path | Df | AIC | BIC | Chisq | Chisq.diff | RMSEA | Df.diff | Pr.  Chisq. |
| --- | --- | --- | --- | --- | --- | --- | --- | --- |
| Adversity T2  – Brain T1 | **6** | 151860 | 152590 | 30.42 | NA | NA | NA | NA |
| Adversity T2  – Brain T1 | **7** | 151858 | 152581 | 30.58 | 0.165 | 0 | 1 | 0.684 |
| Adversity T2  – B. & E. Issues T1 | **6** | 151860 | 152590 | 30.42 | NA | NA | NA | NA |
| Adversity T2  – B. & E. Issues T1 | **7** | 151859 | 152582 | 31.63 | 1.218 | 0.006 | 1 | 0.269 |
| Adversity T3  – Brain T2 | **6** | 151860 | 152590 | 30.42 | NA | NA | NA | NA |
| Adversity T3  – Brain T2 | **7** | 151858 | 152581 | 30.78 | 0.367 | 0 | 1 | 0.544 |
| Adversity T3  – B. & E. Issues T2 | **6** | 151860 | 152590 | 30.42 | NA | NA | NA | NA |
| Adversity T3  – B. & E. Issues T2 | **7** | 151860 | 152583 | 32.32 | 1.902 | 0.013 | 1 | 0.167 |
| Brain T2  - Adversity T1 | **6** | 151860 | 152590 | 30.42 | NA | NA | NA | NA |
| Brain T2  - Adversity T1 | **7** | 151858 | 152581 | 30.65 | 0.234 | 0 | 1 | 0.627 |
| Brain T2  - B. & E. Issues T1 | **6** | 151860 | 152590 | 30.42 | NA | NA | NA | NA |
| Brain T2  - B. & E. Issues T1 | **7** | 151859 | 152582 | 31.54 | 1.128 | 0.005 | 1 | 0.288 |
| Brain T3  - Adversity T2 | **6** | 151860 | 152590 | 30.42 | NA | NA | NA | NA |
| Brain T3  - Adversity T2 | **7** | 151858 | 152581 | 30.48 | 0.062 | 0 | 1 | 0.802 |
| Brain T3  - B. & E. Issues T2 | **6** | 151860 | 152590 | 30.42 | NA | NA | NA | NA |
| Brain T3  - B. & E. Issues T2 | **7** | 151858 | 152581 | 30.76 | 0.342 | 0 | 1 | 0.558 |
| B. & E. Issues T2  - Adversity T1 | **6** | 151860 | 152590 | 30.42 | NA | NA | NA | NA |
| B. & E. Issues T2  - Adversity T1 | **7** | 151860 | 152583 | 32.58 | 2.161 | 0.015 | 1 | 0.141 |
| B. & E. Issues T2  - Brain T1 | **6** | 151860 | 152590 | 30.42 | NA | NA | NA | NA |
| B. & E. Issues T2  - Brain T1 | **7** | 151860 | 152583 | 32.66 | 2.239 | 0.016 | 1 | 0.134 |
| B. & E. Issues T3  - Adversity T2 | **6** | 151860 | 152590 | 30.42 | NA | NA | NA | NA |
| B. & E. Issues T3  - Adversity T2 | **7** | 151858 | 152581 | 30.42 | 0.003 | 0 | 1 | 0.950 |
| B. & E. Issues T3  - Brain T2 | **6** | 151860 | 152590 | 30.42 | NA | NA | NA | NA |
| B. & E. Issues T3  - Brain T2 | **7** | 151858 | 152581 | 30.57 | 0.155 | 0 | 1 | 0.693 |

**Table 3**

*Multigroup results of constrained paths for family conflict and gender. B. & E. Issues = Behavioural and emotional issues.*

| Constrained Cross-Lagged Path | Df | AIC | BIC | Chisq | Chisq.diff | RMSEA | Df.diff | Pr.  Chisq. |
| --- | --- | --- | --- | --- | --- | --- | --- | --- |
| Adversity T2  – Brain T1 | **6** | 151231 | 151961 | 27.73 | NA | NA | NA | NA |
| Adversity T2  – Brain T1 | **7** | 151229 | 151952 | 27.95 | 0.214 | 0 | 1 | 0.643 |
| Adversity T2  – B. & E. Issues T1 | **6** | 151231 | 151961 | 27.73 | NA | NA | NA | NA |
| Adversity T2  – B. & E. Issues T1 | **7** | 151229 | 151952 | 27.74 | 0.004 | 0 | 1 | 0.946 |
| Adversity T3  – Brain T2 | **6** | 151231 | 151961 | 27.73 | NA | NA | NA | NA |
| Adversity T3  – Brain T2 | **7** | 151235 | 151958 | 33.75 | 6.014 | 0.032 | 1 | **0.014** |
| Adversity T3  – B. & E. Issues T2 | **6** | 151231 | 151961 | 27.73 | NA | NA | NA | NA |
| Adversity T3  – B. & E. Issues T2 | **7** | 151229 | 151952 | 27.91 | 0.174 | 0 | 1 | 0.676 |
| Brain T2  - Adversity T1 | **6** | 151231 | 151961 | 27.73 | NA | NA | NA | NA |
| Brain T2  - Adversity T1 | **7** | 151234 | 151956 | 32.45 | 4.716 | 0.028 | 1 | **0.029** |
| Brain T2  - B. & E. Issues T1 | **6** | 151231 | 151961 | 27.73 | NA | NA | NA | NA |
| Brain T2  - B. & E. Issues T1 | **7** | 151229 | 151952 | 27.74 | 0.010 | 0 | 1 | 0.919 |
| Brain T3  - Adversity T2 | **6** | 151231 | 151961 | 27.73 | NA | NA | NA | NA |
| Brain T3  - Adversity T2 | **7** | 151231 | 151954 | 29.97 | 2.235 | 0.016 | 1 | 0.134 |
| Brain T3  - B. & E. Issues T2 | **6** | 151231 | 151961 | 27.73 | NA | NA | NA | NA |
| Brain T3  - B. & E. Issues T2 | **7** | 151230 | 151953 | 28.71 | 0.980 | 0 | 1 | 0.322 |
| B. & E. Issues T2  - Adversity T1 | **6** | 151231 | 151961 | 27.73 | NA | NA | NA | NA |
| B. & E. Issues T2  - Adversity T1 | **7** | 151229 | 151952 | 28.25 | 0.516 | 0 | 1 | 0.472 |
| B. & E. Issues T2  - Brain T1 | **6** | 151231 | 151961 | 27.73 | NA | NA | NA | NA |
| B. & E. Issues T2  - Brain T1 | **7** | 151229 | 151952 | 27.99 | 0.260 | 0 | 1 | 0.609 |
| B. & E. Issues T3  - Adversity T2 | **6** | 151231 | 151961 | 27.73 | NA | NA | NA | NA |
| B. & E. Issues T3  - Adversity T2 | **7** | 151230 | 151953 | 28.62 | 0.888 | 0 | 1 | 0.345 |
| B. & E. Issues T3  - Brain T2 | **6** | 151231 | 151961 | 27.73 | NA | NA | NA | NA |
| B. & E. Issues T3  - Brain T2 | **7** | 151229 | 151952 | 27.74 | 0.011 | 0 | 1 | 0.913 |

**Table 4**

*Multigroup results of constrained paths for neighborhood safety and peers. B. & E. Issues = Behavioural and emotional issues.*

| Constrained Cross-Lagged Path | Df | AIC | BIC | Chisq | Chisq.diff | RMSEA | Df.diff | Pr.  Chisq. |
| --- | --- | --- | --- | --- | --- | --- | --- | --- |
| Adversity T2  – Brain T1 | **6** | 154588 | 155318. | 15.21 | NA | NA | NA | NA |
| Adversity T2  – Brain T1 | **7** | 154586 | 155309 | 15.55 | 0.337 | 0 | 1 | 0.561 |
| Adversity T2  – B. & E. Issues T1 | **6** | 154588 | 155318 | 15.21 | NA | NA | NA | NA |
| Adversity T2  – B. & E. Issues T1 | **7** | 154596 | 155318 | 25.03 | 9.818 | 0.043 | 1 | **0.001** |
| Adversity T3  – Brain T2 | **6** | 154588 | 155318 | 15.21 | NA | NA | NA | NA |
| Adversity T3  – Brain T2 | **7** | 154587 | 155310 | 16.81 | 1.598 | 0.011 | 1 | 0.206 |
| Adversity T3  – B. & E. Issues T2 | **6** | 154588 | 155318 | 15.21 | NA | NA | NA | NA |
| Adversity T3  – B. & E. Issues T2 | **7** | 154589 | 155312 | 18.23 | 3.016 | 0.020 | 1 | 0.082 |
| Brain T2  - Adversity T1 | **6** | 154588 | 155318 | 15.21 | NA | NA | NA | NA |
| Brain T2  - Adversity T1 | **7** | 154586 | 155309 | 15.22 | 0.003 | 0 | 1 | 0.951 |
| Brain T2  - B. & E. Issues T1 | **6** | 154588 | 155318 | 15.21 | NA | NA | NA | NA |
| Brain T2  - B. & E. Issues T1 | **7** | 154588 | 155311 | 17.16 | 1.944 | 0.014 | 1 | 0.163 |
| Brain T3  - Adversity T2 | **6** | 154588 | 155318 | 15.21 | NA | NA | NA | NA |
| Brain T3  - Adversity T2 | **7** | 154587 | 155310 | 16.07 | 0.855 | 0 | 1 | 0.354 |
| Brain T3  - B. & E. Issues T2 | **6** | 154588 | 155318 | 15.21 | NA | NA | NA | NA |
| Brain T3  - B. & E. Issues T2 | **7** | 154586 | 155309 | 15.55 | 0.337 | 0 | 1 | 0.560 |
| B. & E. Issues T2  - Adversity T1 | **6** | 154588 | 155318 | 15.21 | NA | NA | NA | NA |
| B. & E. Issues T2  - Adversity T1 | **7** | 154589 | 155312 | 18.77 | 3.561 | 0.023 | 1 | 0.059 |
| B. & E. Issues T2  - Brain T1 | **6** | 154588 | 155318 | 15.21 | NA | NA | NA | NA |
| B. & E. Issues T2  - Brain T1 | **7** | 154589 | 155311 | 17.90 | 2.690 | 0.018 | 1 | 0.100 |
| B. & E. Issues T3  - Adversity T2 | **6** | 154588 | 155318 | 15.21 | NA | NA | NA | NA |
| B. & E. Issues T3  - Adversity T2 | **7** | 154586 | 155309 | 15.46 | 0.244 | 0 | 1 | 0.621 |
| B. & E. Issues T3  - Brain T2 | **6** | 154588 | 155318 | 15.21 | NA | NA | NA | NA |
| B. & E. Issues T3  - Brain T2 | **7** | 154586 | 155309 | 15.25 | 0.039 | 0 | 1 | 0.841 |

**Table 5**

*Multigroup results of constrained paths for neighborhood safety and gender. B. & E. Issues = Behavioural and emotional issues.*

| Constrained Cross-Lagged Path | Df | AIC | BIC | Chisq | Chisq.diff | RMSEA | Df.diff | Pr. Chisq. |
| --- | --- | --- | --- | --- | --- | --- | --- | --- |
| Adversity T2  – Brain T1 | **6** | 154010 | 154740 | 12.36 | NA | NA | NA | NA |
| Adversity T2  – Brain T1 | **7** | 154009 | 154732 | 13.27 | 0.913 | 0 | 1 | 0.339 |
| Adversity T2  – B. & E. Issues T1 | **6** | 154010 | 154740 | 12.36 | NA | NA | NA | NA |
| Adversity T2  – B. & E. Issues T1 | **7** | 154009 | 154731 | 12.51 | 0.154 | 0 | 1 | 0.694 |
| Adversity T3  – Brain T2 | **6** | 154010 | 154740 | 12.36 | NA | NA | NA | NA |
| Adversity T3  – Brain T2 | **7** | 154009 | 154732 | 12.64 | 0.278 | 0 | 1 | 0.597 |
| Adversity T3  – B. & E. Issues T2 | **6** | 154010 | 154740 | 12.36 | NA | NA | NA | NA |
| Adversity T3  – B. & E. Issues T2 | **7** | 154009 | 154732 | 12.60 | 0.243 | 0 | 1 | 0.621 |
| Brain T2  - Adversity T1 | **6** | 154010 | 154740 | 12.36 | NA | NA | NA | NA |
| Brain T2  - Adversity T1 | **7** | 154009 | 154731 | 12.46 | 0.095 | 0 | 1 | 0.757 |
| Brain T2  - B. & E. Issues T1 | **6** | 154010 | 154740 | 12.36 | NA | NA | NA | NA |
| Brain T2  - B. & E. Issues T1 | **7** | 154008 | 154731 | 12.36 | 0.003 | 0 | 1 | 0.954 |
| Brain T3  - Adversity T2 | **6** | 154010 | 154740 | 12.36 | NA | NA | NA | NA |
| Brain T3  - Adversity T2 | **7** | 154009 | 154731 | 12.47 | 0.108 | 0 | 1 | 0.741 |
| Brain T3  - B. & E. Issues T2 | **6** | 154010 | 154740 | 12.36 | NA | NA | NA | NA |
| Brain T3  - B. & E. Issues T2 | **7** | 154009 | 154732 | 13.19 | 0.829 | 0 | 1 | 0.362 |
| B. & E. Issues T2  - Adversity T1 | **6** | 154010 | 154740 | 12.36 | NA | NA | NA | NA |
| B. & E. Issues T2  - Adversity T1 | **7** | 154009 | 154732 | 12.85 | 0.484 | 0 | 1 | 0.486 |
| B. & E. Issues T2  - Brain T1 | **6** | 154010 | 154740 | 12.36 | NA | NA | NA | NA |
| B. & E. Issues T2  - Brain T1 | **7** | 154009 | 154731 | 12.45 | 0.088 | 0 | 1 | 0.765 |
| B. & E. Issues T3  - Adversity T2 | **6** | 154010 | 154740 | 12.36 | NA | NA | NA | NA |
| B. & E. Issues T3  - Adversity T2 | **7** | 154009 | 154731 | 12.55 | 0.185 | 0 | 1 | 0.666 |
| B. & E. Issues T3  - Brain T2 | **6** | 154010 | 154740 | 12.36 | NA | NA | NA | NA |
| B. & E. Issues T3  - Brain T2 | **7** | 154009 | 154731 | 12.39 | 0.025 | 0 | 1 | 0.872 |
